# Retropharyngeal Internal Carotid Artery Stenosis: A Case-Based Narrative Review

**DOI:** 10.3390/jcm15072683

**Published:** 2026-04-02

**Authors:** Chiara Caruso, Paolo Verlato, Omar Odeh, Roberta Munao, Alessandro Rossi, Besjona Puta, Massimiliano Martelli, Alberto Maria Settembrini

**Affiliations:** 1 Division of Vascular Surgery, IRCCS MultiMedica, Sesto San Giovanni, 20099 Milan, Italy; chiara.caruso@unimi.it (C.C.); paolo.verlato@multimedica.it (P.V.); omar.odeh@multimedica.it (O.O.); roberta.munao@multimedica.it (R.M.); alessandro.rossi@multimedica.it (A.R.); puta.besjona@multimedica.it (B.P.); massimiliano.martelli@multimedica.it (M.M.); 2 School of Vascular Surgery, Università degli Studi di Milano, 20122 Milan, Italy; 3 UniCamillus-Saint Camillus International, Department of Vascular Surgery, University of Health Sciences, 00118 Rome, Italy

**Keywords:** retropharyngeal carotid artery, kissing carotids, endarterectomy, carotid artery stenting, dynamic compression

## Abstract

**Introduction:** The retropharyngeal carotid artery (RCA) is a rare anatomical variant where the carotid artery resides in the retropharyngeal space. The co-occurrence of RCA and significant atherosclerotic stenosis of the carotid bifurcation is even rarer. Recognizing this anatomy is crucial because of the increased risk of adverse events during procedures such as intubation or oropharyngeal surgery. Furthermore, differentiating between the fixed and dynamic forms is essential for guiding appropriate diagnostic and therapeutic strategies. A scoping review was undertaken, and two cases of RCA and significant internal carotid artery stenosis requiring a surgical approach were presented. **Materials and Methods:** EMBASE and OVID were systematically searched for studies reporting data on RCA and significant internal carotid artery stenosis. The Preferred Reporting Items for Systematic Reviews and Meta-Analyses Extension for Scoping Reviews (PRISMA-ScR) was followed, and we presented two case reports of RCA and significant internal carotid artery stenosis requiring surgical treatment, treated at the Division of Vascular Surgery, IRCCS MultiMedica, Sesto San Giovanni, Milan, Italy. **Results and Discussion:** Among the 22 papers identified by the scoping review, 6 case reports were ultimately included in the analysis, supplemented by our two cases. The review and the added cases highlight significant heterogeneity in the clinical presentation and management of RCA with stenosis. Therapeutic options include carotid endarterectomy (CEA), transfemoral carotid artery stenting (TF-CAS), and transcarotid artery revascularization (TCAR). Also, the diagnostic with dynamic 3D-CT angiography during swallowing would be important in some symptomatic cases to document mechanical compression by the hyoid bone or thyroid cartilage (dynamic RCA), which standard static imaging failed to detect. **Conclusions:** Due to the rarity of the condition, no high-level evidence (RCTs) exists. Treatment decisions are based on the qualitative assessment of anatomical risk and isolated case reports. Standard interventions (CEA and TF-CAS) are generally considered high-risk. The final management choice must be individualized based on technical feasibility, neurological risk, and the determination of whether the pathology is fixed or dynamically compressive.

## 1. Introduction

Internal carotid artery (ICA) stenosis remains a leading cause of ischemic cerebrovascular events and is classically encountered at the carotid bifurcation, where diagnostic algorithms and treatment strategies are well established. In contrast, stenotic disease involving atypical anatomical courses of the ICA is uncommon and continues to challenge standard approaches to diagnosis, risk stratification, and management.

A retropharyngeal carotid artery (RCA) represents a rare anatomical variant in which the carotid artery deviates medially and resides within the retropharyngeal space. This configuration is estimated to occur in approximately 2.6% of the general population and is thought to arise from positional changes of the carotid artery during a relatively short window of embryological development [1,2]. Although often incidental, this variant becomes clinically relevant when associated with pathological conditions such as luminal stenosis.

The retropharyngeal location of the ICA carries specific clinical implications. Its close proximity to the pharyngeal wall renders the vessel vulnerable during procedures involving the upper aerodigestive tract, and adverse events have been reported in association with endotracheal intubation and oropharyngeal surgery [3]. When significant atherosclerotic stenosis develops in this setting, the condition assumes a dual significance, combining cerebrovascular risk with an increased potential for iatrogenic injury.

Clinically, retropharyngeal ICA stenosis may be asymptomatic or present with nonspecific symptoms, including dysphagia, globus sensation, or pharyngeal discomfort, and is frequently identified incidentally on cross-sectional imaging performed for unrelated indications. However, the atypical course and orientation of the vessel may complicate both diagnosis and therapeutic decision-making, as conventional criteria derived from typical cervical ICA disease may not be directly applicable.

From a therapeutic standpoint, the management of retropharyngeal ICA stenosis is particularly challenging. Surgical exposure may be technically demanding due to the deep and medial position of the vessel, while endovascular approaches must account for vessel tortuosity, orientation, and the proximity of critical surrounding structures. As a result, treatment strategies are often individualized, reflecting the limited evidence base, which consists largely of isolated case reports and small case series.

In this article, we report two cases of retropharyngeal carotid artery associated with significant internal carotid artery stenosis requiring surgical treatment. In addition, we performed a scoping review of the literature to summarize current knowledge regarding this uncommon clinical scenario. Written informed consent for the publication of clinical data and imaging was obtained from both patients.

## 2. Materials and Methods

This study combined the description of two clinical cases with a scoping review of the literature aimed at summarizing the available evidence regarding the management of internal carotid artery stenosis associated with a retropharyngeal carotid artery (RCA).

The scoping review was conducted in accordance with the Preferred Reporting Items for Systematic Reviews and Meta-Analyses Extension for Scoping Reviews (PRISMA-ScR) guidelines (Supplementary Table S4). The purpose of the review was to identify and summarize published reports describing patients with retropharyngeal internal carotid arteries associated with carotid stenosis and to analyze the diagnostic approaches and therapeutic strategies adopted in these cases.

A comprehensive literature search was performed using the Embase and Ovid databases, including studies published from January 1995 to December 2025. The search strategy was designed to capture studies describing anatomical variants of the internal carotid artery involving the retropharyngeal space, as well as those reporting carotid stenosis in this anatomical configuration. The search strings included combinations of keywords and Medical Subject Headings (MeSH) terms such as retropharyngeal carotid artery, aberrant carotid artery, kissing carotids, internal carotid artery stenosis, carotid endarterectomy, and carotid artery stenting. The complete search strategy and research questions are reported in Supplementary Table S1.

Two independent investigators screened the retrieved studies in a stepwise process. In the first phase, titles and abstracts were reviewed to identify potentially relevant articles. In the second phase, the full texts of the selected studies were assessed for eligibility according to predefined inclusion criteria. Disagreements between the two reviewers were resolved through discussion, and when consensus could not be reached, a third author was consulted to provide a final decision.

The inclusion criteria were studies reporting patients with a retropharyngeal course of the internal carotid artery associated with carotid stenosis, including case reports, case series, or observational studies. Only articles published in English and providing sufficient clinical and procedural details were considered eligible. Studies describing retropharyngeal carotid arteries without stenosis, purely anatomical descriptions, and review articles without original patient data were excluded.

For each included study, relevant data were extracted and organized in a standardized data collection sheet. Extracted variables included patient demographics, clinical presentation, type and degree of carotid stenosis, imaging modalities used for diagnosis, anatomical characteristics of the carotid course, treatment strategy, and procedural outcomes.

Given the rarity of the condition and the expected predominance of case reports, a formal quality assessment of the included studies was not performed, in accordance with PRISMA-ScR recommendations for exploratory scoping reviews.

In addition to the literature review, we report two clinical cases treated at the Division of Vascular Surgery, IRCCS MultiMedica, Sesto San Giovanni, Milan, Italy, in May and September 2020, respectively. Both patients underwent a complete diagnostic evaluation, including duplex ultrasound and computed tomography angiography (CTA), to define the anatomical characteristics of the carotid arteries and the severity of the stenosis. Treatment strategies were discussed within the multidisciplinary vascular team, taking into account symptom status, plaque morphology, and the anatomical feasibility of both surgical and endovascular approaches.

## 3. Results

**Figure 1:**
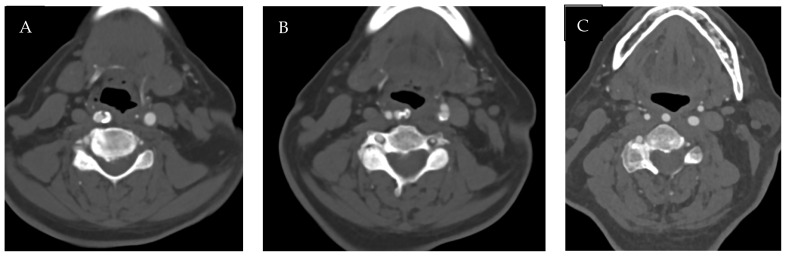
Computed tomography angiography images: (**A**) Retropharyngeal course of the right common carotid artery. (**B**) Right internal carotid artery stenosis with retropharyngeal course. (**C**) Post-stenotic right internal carotid artery with retropharyngeal course.

**Figure 2:**
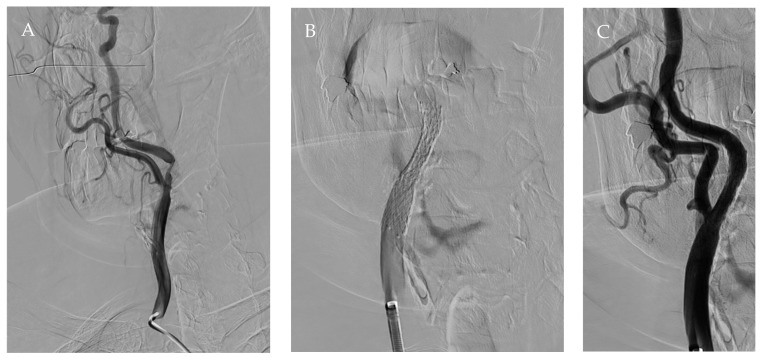
Angiography images: (**A**) Retropharyngeal course of the right common carotid artery during intraoperative angiography, where we observed internal carotid artery stenosis. (**B**) Carotid stent placement. (**C**) Final angiography post-stent placement.

The first case is a 67-year-old diabetic woman with asymptomatic critical (75% with PSV > 300 cm/sec) stenosis of the right internal carotid artery (ICA) with a retropharyngeal course, documented by computed tomography (CT) angiography (Figure 1), who came to our outpatient clinic. The carotid stenosis was an incidental finding during a routine check-up for the diabetic patient, identified via duplex ultrasound, which was challenging to perform due to the unknown anatomical variant. Considering the morphology of the plaque and the anatomical situation, a right carotid artery stenting (CAS) was planned. Under local anesthesia after an epifilter deployment, a CGuard 8 × 40 mm (Inspire MD) stent was placed by a percutaneous right transfemoral approach according to a standard procedure (Figure 2). The postoperative course was regular, and the patient was discharged on the first postoperative day with no complications [4].

**Figure 3:**
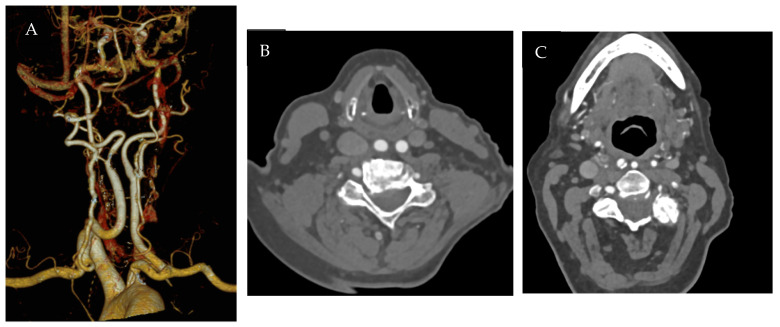
Computed tomography angiography images: (**A**) Retropharyngeal course of the internal carotid artery with 3D reconstruction. (**B**) Kissing carotids is the term used to identify an anatomical variant where two carotid arteries are located very close to each other on the midline. (**C**) Stenosis of the right internal carotid artery with a retropharyngeal course.

The second case is a 73-year-old female patient with symptomatic ICA stenosis of the right internal carotid artery due to a hypoechoic plaque. The patient presented to our emergency room with left upper limb paralysis. CT image reconstructions showed tortuous carotids with a retropharyngeal course. The term commonly used to describe this rare anatomical configuration is ’kissing carotids’, as both carotids are located close to the midline (Figure 3). A CAS was planned, but the procedure proved unsuccessful due to the inability to cross the tortuosity of the supra-aortic vessels and the need to ensure good stability of the devices. A carotid endarterectomy was therefore performed with the support of the otolaryngologist surgeon. Carotid artery exposure was performed according to the Paul André approach, and carotid endarterectomy by eversion technique was performed. The postoperative course was uneventful, and the patient was discharged on the second postoperative day without focal neurological deficits [4].

## 4. Scoping Review

The initial literature search identified 22 potentially relevant publications. After removal of duplicates and screening of titles and abstracts, a subset of articles was selected for full-text evaluation. Following the application of the inclusion and exclusion criteria, six studies were ultimately included in the analysis (Supplementary Figure S1).

Overall, the included studies described six individual patients presenting with a retropharyngeal course of the internal carotid artery associated with carotid stenosis. Detailed characteristics of these cases are summarized in Supplementary Table S2.

The mean age of the patients reported in the literature was 77 years (range 70–82 years). In the majority of cases, the carotid stenosis was symptomatic, with five out of six patients presenting with neurological manifestations such as transient ischemic attack or ischemic stroke. In only one case, the stenosis was detected incidentally during imaging performed for other clinical indications (Table 1).

Different therapeutic approaches were described in the reviewed reports, reflecting the heterogeneity of both anatomical presentation and operator preference. Endovascular treatment was the most frequently adopted strategy. Specifically, 2 patients underwent transcarotid artery stenting, while 2 patients were treated with transfemoral carotid artery stenting. In one case, carotid endarterectomy was performed, while another report described an unusual situation in which resection of the hyoid bone was required to address mechanical compression of the carotid artery.

These findings highlight the absence of a standardized treatment algorithm for this rare anatomical condition. Instead, therapeutic decisions appear to be guided primarily by the individual anatomical configuration of the carotid arteries, the presence or absence of symptoms, and the feasibility of different revascularization techniques.

The small number of reported cases also underscores the rarity of this clinical scenario and the limited level of evidence currently available in the literature. Most reports consist of isolated clinical observations rather than systematic studies, making it difficult to draw definitive conclusions regarding the optimal management strategy.

## 5. Discussion

A retropharyngeal internal carotid artery is an uncommon but important variant that increases the risk of catastrophic bleeding during pharyngeal surgery or intubation. Some authors report conditions in which the anatomical position pushed the operators to a conservative approach [5,6], especially if the carotid stenosis was not symptomatic. On the other side, the presence of symptoms could change the conservative approach to a surgical or endovascular treatment of the stenosis. It was a common characteristic of most cases reported in the literature, suggesting that the peculiarity of the carotid course may be related to the presence of symptoms.

In our series of patients with asymptomatic stenosis, CAS was the treatment of choice due to its minimally invasive nature, considering the complexity of the neck zone of the carotid course and its technical success in a retropharyngeal carotid artery. In the patient with symptomatic stenosis, the critical anatomical complexity (kissing carotids and tortuosity) led to CAS failure, making CEA the only effective and safe revascularization option, with the added benefit of removing a high-risk plaque.

The reports reviewed in the literature highlight the crucial heterogeneity in both pathological presentation and therapeutic strategies, underscoring the lack of a unified treatment consensus.

RCA presents clinical challenges that extend beyond simple fixed atherosclerotic stenosis, as illustrated by some papers, which address the management of high-grade stenosis in a fixed RCA—a primary challenge in routine treatment [3,4,5,6,7].

Ogata et al. introduced another peculiar and rare dynamic condition of a “wandering carotid artery,” characterized by repeated migration of the vessel into the retropharyngeal space, showing that the right internal carotid artery (ICA) moved medially into the retropharyngeal space during CAS, returned to its original position a day after CAS, and had moved medially again into the retropharyngeal space five days after CAS [2]. Yamaguchi et al. described stroke and retinal ischemia caused by thromboembolism induced by dynamic and mechanical compression of the ICA by the hyoid bone and thyroid cartilage during swallowing [8]. This scenario rendered standard diagnostics inadequate. Emphasis was placed on the use of dynamic 3D-CT angiography during swallowing, a crucial method that enabled visualization of the compressive etiology that static imaging could not detect. Suematsu et al. solved the dynamic obstruction leading to recurrent embolic stroke with surgical transposition of the ICA to its normal position [9]. Gates et al. reported recurrent strokes during swallowing in the presence of mild carotid stenosis but with a vulnerable plaque, focusing on the primary etiological mechanism (vulnerable plaque) despite the mild degree of stenosis as the source of emboli [10]. Dynamic compression acts as a “crushing” force, destabilizing the plaque’s surface and releasing embolic material.

It is therefore fundamental to differentiate between fixed RCA and dynamic RCA (wandering or compressive). Dynamic diagnostics (e.g., dynamic CT angiography during swallowing) should be considered in the presence of symptoms suggestive of compression (e.g., dysphagia) or when the ischemic etiology is unclear, preventing the potential underestimation of the pathology by standard techniques (static CT/MRA) (Mechanical Compression of the Carotid Artery by the Pharynx in the Retropharyngeal Space During Swallowing and the Induction of Stenosis and Embolic Stroke: Illustrative Case, 2023).

The treatment of stenosis in the presence of RCA is complex, with most authors agreeing that traditional carotid endarterectomy (CEA) is associated with a high risk due to difficulty in anatomical exposure, the potential for cranial nerve injury, and an increased risk of periprocedural stroke [2,3,7].

Shennib and Ettleson proposed transcarotid artery revascularization (TCAR) as a safer alternative to both CEA and transfemoral CAS (TF-CAS) for aberrant anatomy, with the goal of minimizing operative and neurological complications [7,11].

Safi et al. opted for TF-CAS to manage asymptomatic stenosis, demonstrating the technical success of an endovascular approach in this anatomical setting [3].

The final therapeutic decision is guided by the assessment of neurological risk and technical feasibility.

Finally, the “tailored” approach by Yamaguchi et al., who performed partial resection of the hyoid bone and thyroid cartilage, reasserts the need for an etiological treatment in cases of documented mechanical compression [8].

It must be acknowledged that several clinically relevant details—such as specific peri-procedural pharmacological protocols, long-term follow-up data, and detailed neurological workups—were inconsistently reported across the reviewed literature. This lack of standardized reporting represents a limitation of the current evidence base and may affect the generalizability of the findings regarding clinical outcomes and long-term vessel patency.

## 6. Conclusions

The extreme rarity of RCA with stenosis means that no randomized controlled trials (RCTs) or systematic reviews of large cohorts exist that can provide quantitative complications, but it is a rare but high-risk anatomical variant that may coexist with or mimic internal carotid artery stenosis. Dynamic imaging is crucial for diagnosis, and management requires individualized planning—favoring conservative treatment or modified surgical approaches when the ICA lies close to the pharynx.

The retropharyngeal internal carotid artery represents a rare anatomical variant that may have important clinical implications, particularly when associated with significant carotid stenosis. Although the condition is often discovered incidentally, its presence may increase the complexity of both diagnostic evaluation and therapeutic management.

The medial displacement of the internal carotid artery within the retropharyngeal space alters the typical anatomical relationships in the cervical region and may expose the vessel to potential injury during pharyngeal or airway procedures. Moreover, when atherosclerotic disease develops in this setting, the abnormal course of the artery may complicate both surgical exposure and endovascular access, requiring careful preoperative planning and individualized treatment strategies.

Our experience, combined with the limited evidence available in the literature, suggests that treatment decisions should be tailored to the specific anatomical and clinical context of each patient. Endovascular approaches may represent a valuable option in selected cases, particularly when surgical exposure is challenging due to the deep and medial position of the artery. However, severe tortuosity, unfavorable supra-aortic anatomy, or unstable catheter positioning may limit the feasibility of carotid artery stenting, making carotid endarterectomy the preferred option in certain patients.

An additional aspect that deserves particular attention is the distinction between fixed retropharyngeal carotid arteries and dynamic or compressive variants, such as wandering carotid arteries or those subjected to mechanical compression by adjacent structures during swallowing. In these situations, dynamic imaging techniques may play a crucial role in identifying the underlying pathophysiological mechanism and guiding appropriate treatment.

Because of the extreme rarity of this condition, the current body of evidence is limited to case reports and small case series, and no randomized controlled trials or large observational studies are available to provide robust comparative data. Consequently, management strategies must rely on careful anatomical assessment, multidisciplinary discussion, and the experience of specialized vascular centers.

Further studies and accumulation of additional case reports are needed to improve the understanding of this rare anatomical variant and to better define the most appropriate diagnostic and therapeutic strategies. Increased awareness among vascular surgeons, radiologists, anesthesiologists, and otolaryngologists is essential in order to prevent potential complications and ensure optimal management of patients presenting with this uncommon but clinically significant condition.

## Figures and Tables

**Table 1:** Summary of patient demographics and management strategies in carotid artery case reports (N = 6 cases).

Case Detail	Pooled Data (N = 6 Cases)
Mean Age (Years)	Range: 70–82
Symptomatic Stenosis	5 cases
Asymptomatic Stenosis	1 case
Transcarotid Stenting (TCAR)	2 cases
Transfemoral Carotid Stenting (CAS)	2 cases
Surgical Removal (Endarterectomy/Resection)	1 case (hyoid bone removal)
Other	1 case (swallowing-triggered)

## Data Availability

No new data were created or analyzed in this study.

## Supplementary Materials

The following supporting information can be downloaded at: https://www.mdpi.com/article/10.3390/jcm15072683/s1, Figure S1: Selection and Exclusion of Identified Studies; Table S1: Research Question and Search Strings; Table S2: Details of Single Selected Paper; Table S3: Symptoms Diagnosis; Table S4: PRISMA-ScR checklist [12].
