# Supplementary material for: Retropharyngeal Internal Carotid Artery Stenosis: A Case-Based Narrative Review
Source: J Clin Med. 2026 Apr 2;15(7):2683. doi: 10.3390/jcm15072683 (PMC13074077; doi:10.3390/jcm15072683)
Supplement: Supplementary file 1 [file jcm-15-02683-s001.zip › Figure S1 - Selection and Exclusion of Identified Studies.pdf]

Supplementary Fig. S1. Selection and Exclusion of Identified Studies

Main Author (Year)	Status	Reason for Inclusion / Exclusion
Ogata (2025)	Included	RCA with symptomatic stenosis treated with CAS.
Shennib (2024)	Included	RCA with symptomatic stenosis treated with TCAR.
Ettleson (2024)	Included	RCA with symptomatic stenosis treated with TCAR.
Yamaguchi (2020)	Included	Dynamic RCA with mechanical compression treated by surgical resection.
Gates (2017)	Included	Symptomatic carotid stenosis triggered by swallowing (CEA).
Martin/Safi (2016)	Included	RCA with asymptomatic high-grade stenosis treated with CAS.
Parhar (2021)	Excluded	Focus on oropharyngeal malignancies; absence of significant carotid stenosis.
Sorour (2021)	Excluded	Managed with Best Medical Therapy (BMT); no surgical or endovascular intervention.
Garrido (2020)	Excluded	Anatomical review for complication prevention; no specific carotid stenosis treated.
Ballivet (2017)	Excluded	Description of an anatomical variant without associated carotid stenosis.
Koreckij (2013)	Excluded	Retrospective cohort study on radiological incidence; no focus on carotid stenosis treatment.
Baba (2017)	Excluded	Focus on "wandering" vessel mobility on CT scan; no significant carotid stenosis reported.
Alzubaidi (2024)	Excluded	Clinical presentation as a pharyngeal mass; focus on surgical tecnique awareness without stenosis.
Abishek (2024)	Excluded	Description of anatomical aberration; absence of carotid stenosis pathology.
Calzolari (1998)	Excluded	Diagnostic radiological study (CT); insufficient clinical or interventional data.
Rohrer (2011)	Excluded	Radiology quiz format; missing specific details on carotid stenosis management.
Abuhalimeh (2022)	Excluded	Incidental finding during intubation; no treatment for carotid stenosis.
Babu (2017)	Excluded	Technical notes on difficult stenting; lack of detailed patient data.
Benson (2023)	Excluded	Study on plaque composition and tortuosity; not specific to the retropharyngeal variant.
Mousa (2013)	Excluded	Clinical description of the variant without focus on carotid stenosis treatment.
