# Supplementary material for: Retropharyngeal Internal Carotid Artery Stenosis: A Case-Based Narrative Review
Source: J Clin Med. 2026 Apr 2;15(7):2683. doi: 10.3390/jcm15072683 (PMC13074077; doi:10.3390/jcm15072683)
Supplement: Supplementary file 1 [file jcm-15-02683-s001.zip › Table S1 - Research Question and Search Strings.pdf]

Supplementary Table S1. Research question and search strings.

Research question	What is the incidence of carotid stenosis in the presence of a retropharyngeal course of the internal carotid artery (ICA)?
Search strings	EMBASE : ('carotid stenosis'/exp OR 'carotid artery stenosis' OR carotid stenosis) AND ('retropharyngeal space'/exp OR retropharyngeal OR retro-pharyngeal)'carotid stenosis'/exp AND 'retropharyngeal abscess'/exp AND [mh "carotid artery, internal"]'carotid stenosis':ab,ti AND 'retropharyngeal':ab,ti<br><br>OVID: (exp Carotid Stenosis/ OR carotid stenosis.mp.) AND (exp Retropharyngeal Space/ OR retropharyngeal.mp. OR retro-pharyngeal.mp.)(Carotid Stenosis/ OR carotid artery stenosis.mp. OR carotid stenosis.mp.) AND (Retropharyngeal Abscess/ OR retropharyngeal.mp.) AND (exp Internal Carotid Artery/)ti,ab(carotid stenosis) AND ti,ab(retropharyngeal)
