# Supplementary material for: Retropharyngeal Internal Carotid Artery Stenosis: A Case-Based Narrative Review
Source: J Clin Med. 2026 Apr 2;15(7):2683. doi: 10.3390/jcm15072683 (PMC13074077; doi:10.3390/jcm15072683)
Supplement: Supplementary file 1 [file jcm-15-02683-s001.zip › Table S2 - Details of Single Selected Paper.pdf]

ID Articolo	Title	Main Authors	Year of publication	Journal	Type of study	Sample	Intervention	Outcome	Symptoms
A001	Wandering Carotid Artery in Carotid Artery Stenting.	Ogata A, Furukawa T, Masuoka J, Abe T	2025	Cureus - Volume 17, Issue 2, pp. e78788	Case report	F, 82 y	CAS	After CAS showed that the ICA had returned to its original position	Symptomatic ICA dx (TIA)
A002	Retropharyngeal carotid artery stenosis; potential for minimizing operative complications utilizing transcarotid artery stent revascularization	Shennib, H., Huerta, V.	2024	J. Vasc. Surg. Cases. Innov. Tech. - Volume 10, Issue 6, pp.	Case report	M, 80 y	TCAR	TCAR may be a safer modality for its treatment.	Symptomatic ICA dx (acute non-hemorrhagic left frontal lobe infarct within the territory of the middle cerebral artery)
A004	Transcarotid Artery Revascularization for Symptomatic Retropharyngeal Internal Carotid Artery Stenosis.	Ettleson, Ari, Robbins, Justin, Ascher, Enrico, Hingorani, Anil	2024	Vascular and endovascular surgery - Volume 58, Issue 8, pp. 884-887	Case report	F, 70 y	TCAR	TCAR may be a safer modality for its treatment.	Symptomatic ICA
A0003	Dynamic 3D-CT angiography during swallowing for diagnosing hyoid bone or thyroid cartilage compression-induced thromboembolism	Yamaguchi, Y., Saito, A., Ohsawa, Y., Nagasawa, H., Wada, M.	2020	Radiol. Case Rep. - Volume 15, Issue 9, pp. 1468-1472	Case report	M, 76 y	mechanical compression of the right ICA by the hyoid bone and thyroid cartilage as a cause of embolic	partial resection of the right greater horn of the hyoid bone and the right superior horn of the thyroid cartilage was performed by otolaryngologists	Visual loss: acute ischemic lesions in the territory of the right middle cerebral artery
A005	Mild Carotid Stenosis with Recurrent Symptoms Triggered by Eating.	Gates, Marcus J, Brinjikji, Waleed, Williams, Lindsy	2017	World neurosurgery - Volume 97, Issue 101528275, pp. 750.e11-750.e13	Case report	M, 78 y	multiple recurrent emboli - TEA ICA	Due to mechanical effect of the esophagus "squeezing"	multiple recurrent strokes and transient ischemic attacks occured with eating
A006	Stenting of a Retropharyngeal Internal Carotid Artery.	Martin, Gordon H, Saqib, Naveed U, Safi, Hazim J	2016	Annals of vascular surgery - Volume 34, Issue 0, pp. 268.e1-3	Case report	F, 76 y	CAS (80-90% stenosis)	the results of carotid stenting for asymptomatic lesions are acceptable, as seen in the recent CREST trial	asymptomatic internal carotid artery stenosis
