# Supplementary material for: Retropharyngeal Internal Carotid Artery Stenosis: A Case-Based Narrative Review
Source: J Clin Med. 2026 Apr 2;15(7):2683. doi: 10.3390/jcm15072683 (PMC13074077; doi:10.3390/jcm15072683)
Supplement: Supplementary file 1 [file jcm-15-02683-s001.zip › Table S3 - Symptoms Diagnosis.pdf]

Article ID	Sex	Age (Years)	Initial Symptoms/Diagnosis
A001	F	82	Symptomatic for TIA (Transient Ischemic Attack).
A002	M	80	Symptomatic for acute ischemic stroke (non-hemorrhagic left frontal lobar infarct within the territory of the middle cerebral artery).
A004	F	70	Symptomatic ICA.
A003	M	76	Acute visual loss; ischemic lesions in the territory of the middle cerebral artery.
A005	M	78	Multiple recurrent ischemic strokes and TIAs triggered by swallowing.
A006	F	76	Asymptomatic internal carotid artery stenosis.
